# Non-Hodgkin’s Lymphoma Presenting as Isolated Peritoneal Lymphomatosis: A Case Report and Literature Review

**DOI:** 10.3389/fonc.2021.719554

**Published:** 2021-09-02

**Authors:** Min Zhu, Zhixuan Wu, Zhaoxia Yang, Bo Ning, Shengjie Yu, Xiling Gu, Huihong Yu

**Affiliations:** ^1^Department of Gastroenterology, The Second Affiliated Hospital of Chongqing Medical University, Chongqing, China; ^2^Department of Urology, The Second Affiliated Hospital of Chongqing Medical University, Chongqing, China; ^3^Department of Pathology, The Second Affiliated Hospital of Chongqing Medical University, Chongqing, China

**Keywords:** peritoneal lymphomatosis, non-Hodgkin’s lymphoma, peritoneal carcinomatosis, diagnosis, case report

## Abstract

Peritoneal lymphomatosis is extremely rare and associated with poor prognosis. Most practitioners only pay more attention to peritoneal carcinomatosis. However, peritoneal lymphomatosis can be neglected and misdiagnosed. We report a teenager with 10 days of abdominal distension and pain accompanied by computed tomography scan suggesting diffuse thickening of the peritoneum and omentum and abdominopelvic effusion. Tuberculous peritonitis and peritoneal carcinomatosis were initially suspected. However, it was finally confirmed as non-Hodgkin’s B-cell lymphoma by omentum biopsies. He achieved complete remission after chemotherapy and autologous stem cell transplantation. But unfortunately, he suffered a relapse and died 10 months after diagnosis. Following a review of the literature, it can be concluded that the discovery of lymphomatosis in peritoneum is a rare finding. Lymphoma should be considered in the differential diagnosis of unexplained peritoneal thickening on computed tomography, and this case emphasizes the importance of early pathological diagnosis to make sure that the right treatment can be started opportunely.

## Introduction

Non-Hodgkin’s lymphoma (NHL) includes more than 90 distinct genotypes of hematologic malignancies. About 90% of NHL cases are of B-cell origin, most commonly seen in diffuse large B-cell lymphoma (DLBCL) (1). DLBCL has been described in any site of the body, most occurring in nodal (usually cervical or abdominal) or extranodal disease, and extranodal involvement occurs in approximately 40% of DLBCLs (2, 3). However, peritoneal lymphomatosis (PL), which receives much less attention than peritoneal carcinomatosis and is more often misdiagnosed, usually related to diffuse B-cell lymphoma, refers to the seeding of parietal peritoneum and surface of the covered abdominal organs with lymphoma cells (4). The most frequent clinical symptoms of peritoneal lymphomatosis are abdominal pain, distension, and weight loss (5). We herein describe a case of a young teenager wherein a clinical picture resembling tuberculous peritonitis and peritoneal carcinomatosis was subsequently diagnosed as non-Hodgkin’s B-cell lymphoma with isolated peritoneal involvement.

## Case Presentation

A 16-year-old boy was admitted to our hospital with a 10-day history of abdominal distension and epigastric pain, accompanied by anorexia, nausea, vomiting, dyspnea, palpitations, as well as low fever and night sweating. He had always been in good health with no significant medical or malignant family history.

On physical examination, the patient showed tachycardia, abdominal tension, tenderness, rebound tenderness in the upper quadrant, and shifting dullness. No palpable masses and no extremity edema were noted. Neither lymphadenopathy nor hepatosplenomegaly was detected.

Peripheral blood counts elevated in white blood cell 10.71×10^9^/L (reference range: 3.50–9.50×10^9^/L), neutrophils 8.03×10^9^/L (reference range: 1.80–6.30×10^9^/L), and monocytes 1.10×10^9^/L (reference range: 0.10–0.60×10^9^/L). The values of lymphocytes, hemoglobin, and platelets were normal. Fecal occult blood test was positive. The laboratory tests revealed a high uric acid level of 1,240.2 μmol/L (reference range: 208.0–506.0 μmol/L), aspartate aminotransferase value of 57 U/L (reference range: 15–46 U/L), and the serum carbohydrate antigen 125 value of 758 U/ml (reference range, <35 U/ml). Other values of blood urea nitrogen, creatinine, electrolytes, Epstein-Barr virus (EBV), human immunodeficiency virus (HIV), as well as other tumor markers (Carcinoembryonic antigen, Carbohydrate antigen 19−9, and Alpha fetoprotein), and hepatitis markers were negative. Assistant examinations associated with tuberculosis (Tuberculin skin test, T-SPOT test, and IgG antibody for tuberculosis) were negative. Additionally, abdominocentesis and ascitic fluid examination were performed as well. Orange-yellow turbid fluid with predominance of mononuclear cell indicated exudate.

**Figure 1:**
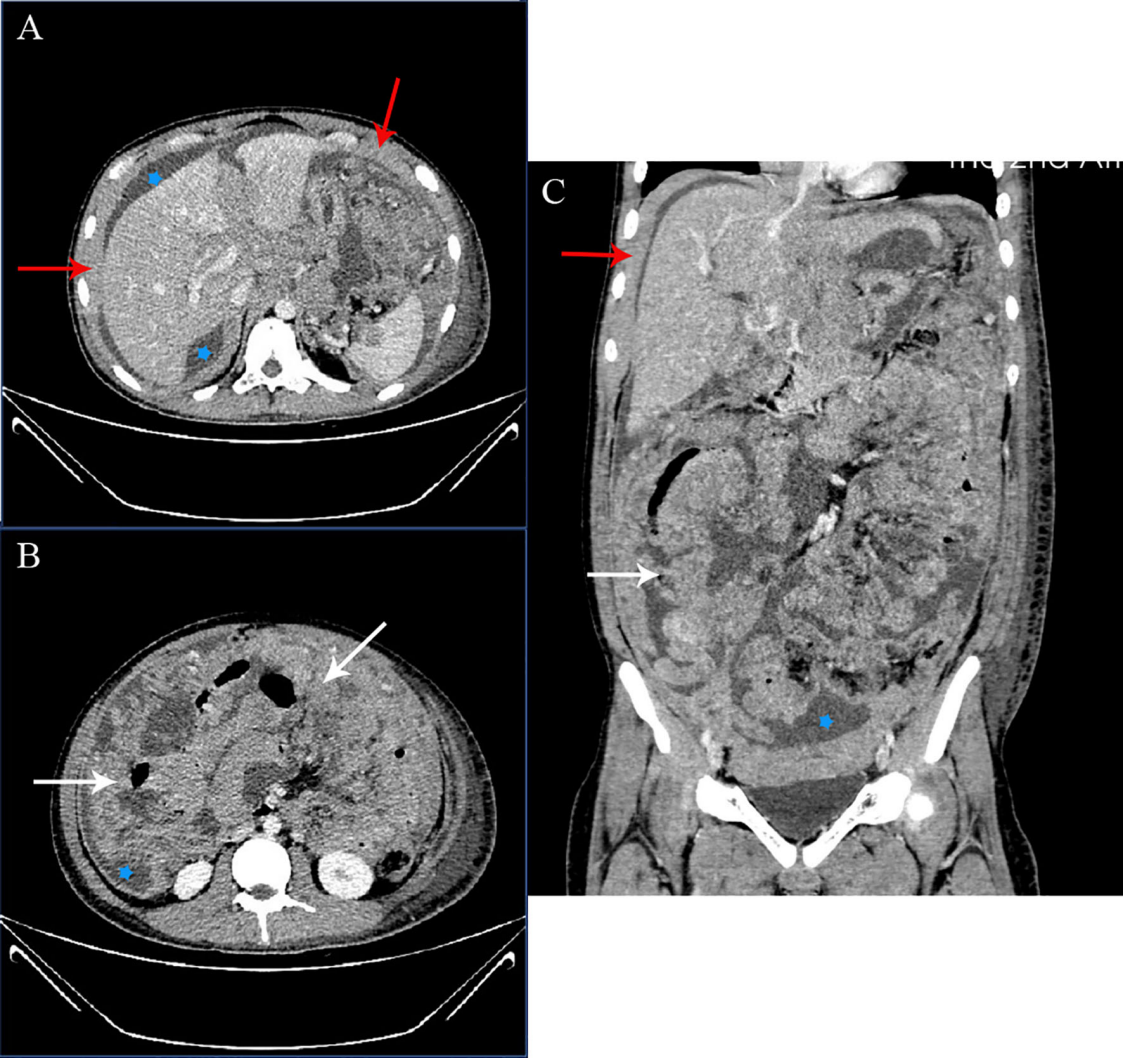
CT examination before-treatment. Axial **(A)**, Axial **(B)**, and coronal **(C)** enhanced CT images of the abdomen and pelvis in the portal phase. Extensive ascites (stars), thickening of the peritoneum (red arrow), and marked thickening of the omentum (white arrows) noted. No enlarged lymph nodes were noted.

**Figure 2:**
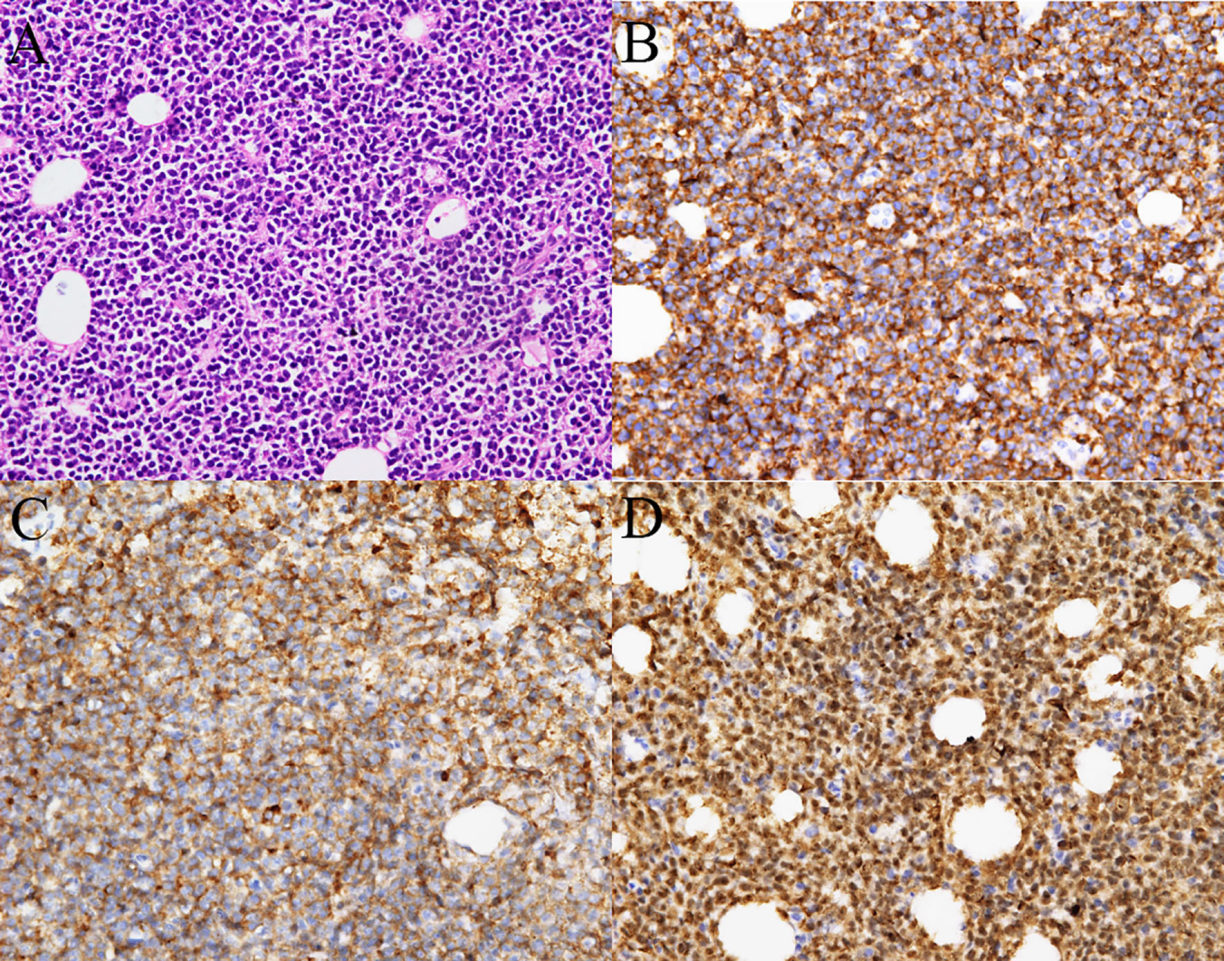
Histopathology from the omental thickening biopsy. **(A)** Hematoxylin-eosin staining showed diffuse infiltration of neoplastic lymphoid cells (400×). Tumor cells were immunoreactive for **(B)** CD20, **(C)** CD10, **(D)** and expressed high Ki-67 index (nearly 90%) (400×).

Cervicothoracic and whole abdominal computed tomography (CT) scan without any intestinal involvement, tumor formation, or enlarged lymph nodes revealed diffuse thickening of peritoneum and omentum, thoracic, abdominal, and pelvic effusion (**Figure 1**), inflammation in the inferior lobe of the right lung, strip shadow of hepatic hilum and hepatogastric space, dilatation of intrahepatic bile duct, as well as edema and thickening of gallbladder wall. During the coronavirus disease 2019 (COVID-19) pandemic, since the patient’s condition deteriorated progressively, we failed to take any gastrointestinal endoscopies. The result of bone marrow biopsy was negative for malignancy. Finally, the patient underwent a surgical laparoscopy to determine the cause. A biopsy of the greater omentum showed diffuse peritoneal thickening and nodular omental infiltration, consistent with an aggressive type of non-Hodgkin’s B-cell lymphoma. Immunohistochemical stain subsequently showed positivity for CD10, CD20, PAX-5, and c-myc with proliferative fraction of 90% (**Figure 2**), negativity for CD3, CD56, CD138, and Mum-1, which was in favor of the diagnosis of diffuse large B-cell lymphoma, germinal center B-cell type, stage IV.

**Figure 3:**
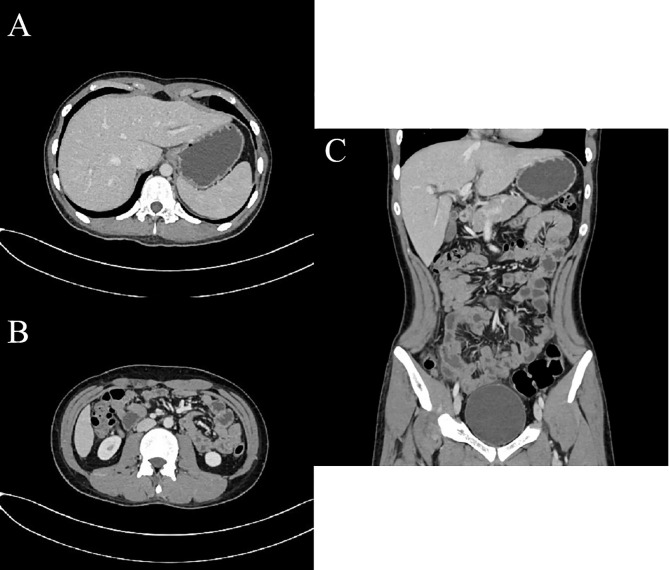
CT examination post-treatment. Axial **(A)**, Axial **(B)**, and coronal **(C)** enhanced CT images of the abdomen and pelvis in the portal phase post-treatment demonstrated resolution of ascites, thickening of peritoneum and omentum disappeared and turned to the normal shape.

Considering the possibility of a diagnosis of peritoneal carcinomatosis, the patient firstly received three courses of hyperthermic intraperitoneal chemotherapy, followed by five cycles of cyclophosphamide, doxorubicin, vincristine, and Prednisone (CHOP) regimen with rituximab. During the first course of chemotherapy, the patient experienced signs and symptoms concerning for tumor lysis syndrome, including oliguria, hyperuricemia, hyperkalemia, hyperphosphatemia, and hypocalcemia. Follow-up CT reexamination demonstrated remission of his condition (**Figure 3**). Later autologous peripheral blood stem cell transplantation was completed 4 months after the chemotherapy. Unfortunately, the patient suffered a relapse 4 months after the blood stem cell transplantation and was given cisplatin, dexamethasone, and high-dose cytarabine (DHAP) regimen with rituximab, administered concurrently with intrathecal chemotherapy (methotrexate, cytarabine, dexamethasone). In the end, the patient suffered severe marrow suppression and gave up all the treatments for financial difficulty and died 10 months after initial diagnosis.

## Discussion

Peritoneal lymphomatosis (PL), which is described and explained in few past cases and studies (seen in **Table 1**), is always found in patients with DLBCL and Burkitt’s lymphoma and more often occurs in males. There is variation in age distribution but found mainly in children and the elderly (4, 5, 11–13). The youngest case reported until now was a 3-year-old infant (14). To our knowledge, this is the first report of isolated peritoneal lymphomatosis with DLBCL in a teenager. Similar to our case, most PL patients predominantly complain of abdominal pain, distension, and weight loss (5, 8, 11), though these clinical manifestations are non-specific.

**Table 1:** Literature review of peritoneal lymphomatosis.

Patient	Sex, age	Details of symptoms	Image findings	Diagnosis	Treatment	Outcome	References
1	Male, 7 y	Abdominal pain, distention, vomiting and melena.	Thickened omentum, intestines, and peritoneum (caking); non-specific supraclavicular lymph nodes.	Omental biopsy: Burkitt’s lymphoma	Appropriate chemotherapy	Significant remission of hepatic nodules, mesenteric, omental, and bowel thickening	(6)
2	Female, 57 y	Abdominal fullness, nausea, vomiting, weakness, malaise, and edema.	Diffusely thickened, nodular, and irregular peritoneum.	Peritoneal biopsy: DLBCL	Not known	Died 4 days after the surgical laparoscopy	(4)
3	Male, 61 y	Progressive increase in abdominal volume and loss of weight.	Diffuse nodular peritoneal thickening with heterogeneous enhancement, thickening of the greater omentum (caking).	Peritoneal biopsy: Burkitt’s lymphoma	COPADM protocol	Not known	(7)
4	Male, 72 y	Abdominal distension, anorexia, weakness, shortness of breath, and weight loss.	Thickened diaphragm and peritoneum with omental cake, ascites, and extensive enlarged lymph nodes.	The enlarged supraclavicular lymph node biopsy: follicular lymphoma	Eight cycles of R-CHOP therapy	Substantial reduction in tumor burden	(8)
5	Male, 29 y	Nausea, vomiting, diarrhea, abdominal pain.	Irregularly thickened wall of stomach with a gastric mass, and extensive nodules on omentum.	Peritoneal biopsy: Burkitt’s lymphoma	The first cycle of R-CHOP therapy, four cycles of Hyper-CVAD/MA therapy	Complete response	(5)
6	Male, 46 y	Abdominal pain and weight loss.	Diffuse peritoneal involvement.	Peritoneal biopsy: DLBCL	Eight cycles of R-CHOP therapy	Complete response	(5)
7	Female, 67 y	Abdominal pain, bloating, loss of appetite and weight, nausea, vomiting, diarrhea.	Probable mesenteric and omental carcinomatosis with an irregular lump.	Peritoneal biopsy: DLBCL	Eight cycles of R-CHOP therapy	Complete response	(5)
8	Female,18 y	Abdominal pain and fullness.	Diffuse thickening of peritoneum and omentum, and nodularity.	Omental and peritoneal biopsy: Burkitt’s lymphoma	R-Hyper-CV AD alternating with intrathecal MTX/Ara-C	Complete response	(9)
9	Female, 65 y	Abdominal distention, anorexia, night sweating, and melena.	Omental cake, ascites, and metastatic lymph nodes.	Ascites cytology and gastric tumor biopsy: gastric DLBCL	Seven cycles of R-CHOP therapy	Died 10 months after diagnosis	(10)
10	Male, 61 y	Abdominal distention, palpitations, dyspnea, and sweating.	Ascites, diffuse thickening of peritoneum, and soft tissue nodularity.	Ultrasound-guided core needle biopsy of peritoneum and omentum: high-grade B-cell lymphoma	First cycle: R-CHOP therapy, subsequent cycles: R-CHOP regimen with dose-adjusted etoposide	Died 4 months after diagnosis	(1)
11	Male, 16 y	Fever, abdominal pain, and distension.	Ascites, thickened and nodular omentum.	Omental and bone marrow biopsies: Burkitt’s lymphoma	CHOP therapy	Died	(11)
12	Male, 16 y	Abdominal distension and pain, anorexia, nausea, vomiting, shortness of breath, palpitations, as well as low-grade fevers and night sweating.	Diffuse thickening of peritoneum and omentum, abdominal and pelvic effusion.	Omentum biopsy: DLBCL	Chemotherapy and autologous peripheral blood stem cell transplantation	Died 10 months after diagnosis	Our patient

DLBCL, diffuse large B-cell lymphoma; COPADM, cyclophosphamide, vincristine, prednisone, doxorubicin, methotrexate; CVAD, cyclophosphamide, vincristine, doxorubicin, dexamethasone; MTX, methotrexate; Ara-C, cytarabine; R-CHOP, rituximab, cyclophosphamide, doxorubicin, vincristine, and prednisone.

There usually are two ways of peritoneal invasion in malignant lymphoma (10). First, primary effusion lymphoma (PEL), an uncommon B-cell malignancy, associated with Kaposi’s sarcoma-associated herpesvirus/Human herpesvirus 8 (KSHV/HHV-8) infection and coinfection with EBV or HIV, most often occurs in immunocompromised patients. The main characteristic of PEL is neoplastic effusions in body cavities without any detectable entity tumor (15–17). Second, the peritoneal affection can occur secondary to lesions in the digestive tract or abdominal lymph nodes (18). Moreover, since the omentum does not contain lymphoid tissue, it is uncommon to affect lymphoma. Though the pathway of dissemination is unclear, the possible routes are gastrocolic ligament, transverse mesocolon, and visceral peritoneal surfaces (12, 19). In our case, we discuss a young teenager presenting as isolated PL mainly considering his CT and follow-up imaging did not detect any evidence suggesting primary bowel and lymph node involvement. According to the classification, it refers to PEL, but the patient had no evidence of immunocompromise and infection of EBV or HIV. A limitation of this study is that our patient didn’t take any endoscopies because of the deterioration of the condition during the COVID-19 pandemic. Therefore, we cannot fully rule out the possibility of primary intestinal lymphoma infiltrating the peritoneum and omentum.

The differential diagnosis of peritoneal thickening includes peritoneal lymphomatosis, peritoneal carcinomatosis, tuberculous peritonitis, mesothelioma, and metastatic tumors, such as ovarian, breast, and gastrointestinal carcinomas (20, 21). PL has common imaging characteristics with peritoneal carcinomatosis and tuberculosis containing ascites, thickened peritoneum and omentum (22). An earlier study reported the distinguishing CT findings of peritoneal lymphomatosis including ascites without any loculation or septation and diffuse enlarged lymph nodes (21). Cabral et al. suggested that no gastrointestinal tract involvement is indicative of peritoneal lymphomatosis and that diffuse lymph node enlargement, smooth peritoneal soft tissue thickening or bulky homogeneous masses, as well as imaging findings of changeable extranodal lymphoma involvement were helpful signs of peritoneal lymphomatosis (19). Imaging findings of lymphomatosis rather than carcinomatosis include lymphadenopathy, large mesenteric mass, and splenomegaly (22). Positron emission tomography computed tomography (PET-CT) scan can be utilized to differentiate whether the ascites are caused by malignant lesions (23). It shows hypermetabolic activity of the peritoneal thickening may contribute to guide sampling, especially in DLBCL and Burkitt’s lymphoma (24, 25). CT/PET-CT may be the first choice to distinguish these kinds of diseases. In our case, as our patient was decided to undergo the omental biopsy, he didn’t take the PET-CT, which limited the certainty of the diagnoses regarding if the disease was isolated to the peritoneum or was a part of a more generalized lymphoma. His CT scan suggested diffuse thickening of the peritoneum and omentum with large-volume ascites, which reflected common imaging features of peritoneal lymphomatosis and peritoneal carcinomatosis. The image of our patient did not have any lymph node enlargement or splenomegaly in favor of lymphomatosis. Moreover, the gastrointestinal tract and digestive organs were normal, so we could not exclude either disease.

As imaging findings are non-specific, it has been proven that only pathology can make a definitive diagnosis (26). Aathira et al. reported a case of peritoneal tuberculosis that was confirmed as Burkitt’s lymphoma by omental and bone marrow biopsies (11). Another study reported a rare presentation of follicular lymphoma simulating peritoneal carcinomatosis, and the biopsy of the enlarged supraclavicular lymph node was reported in keeping with follicular lymphoma (8). Besides, histology is regarded as good criteria of diagnosis of peritoneal lymphomatosis, because cytology is not always available and lymphoma can elicit florid mesothelial hyperplasia, leading to inaccurate results and lengthening the diagnostic process (27, 28). Taking into account the history, the imaging findings, and the ascites analysis, the initial possibilities of our case included peritoneal tuberculosis and peritoneal carcinomatosis. Surprisingly, we took prompt omentum biopsies, and it was finally confirmed as non-Hodgkin’s B-cell lymphoma.

Peritoneal lymphomatosis is rare and aggressive. Based on these previous experiences and literature reviews, chemotherapy regimens for peritoneal lymphoma are similar to those for DLBCL using CHOP with or without rituximab based on staging (5, 10). Twenty-five percent of cases with peritoneal lymphomatosis were reported to have a good response to chemotherapy and prognosis (11, 29). Conversely, DLBCL behaves aggressively and has a poor prognosis. If untreated, the median survival is less than 1 year (30). It is therefore necessary to recognize this rare disease to obtain a pathological diagnosis as early as possible and make sure that the correct treatment can be started opportunely.

In conclusion, PL is a rare presentation of extranodal involvement of lymphoma with known poor prognosis. With no characteristic clinical presentations and specific imaging, PL is often misdiagnosed, and the delay of the diagnosis reduces the possibility for optimal treatment and chance of survival. In such ambiguous cases, early histological diagnosis is the primary tool of the rapid and effective treatment.

## Data Availability Statement

The original contributions presented in the study are included in the article/supplementary material. Further inquiries can be directed to the corresponding authors.

## Ethics Statement

Ethical review and approval was not required for the study on human participants in accordance with the local legislation and institutional requirements. Written informed consent to participate in this study was provided by the participants’ legal guardian/next of kin. Written informed consent was obtained from the individual(s), and minor(s)’ legal guardian/next of kin, for the publication of any potentially identifiable images or data included in this article.

## Author Contributions

MZ did the data collection and wrote the original draft. ZW, ZY, BN, SY, and HY contributed to patient’s care and did a critical revision of the manuscript. XG contributed to the pathology description and images. HY and XG contributed to the final version of the manuscript. All authors contributed to the article and approved the submitted version.

## Funding

This work was supported by Kuanren Talents Program of the Second Affiliated Hospital of Chongqing Medical University and Young Medical High-end Reserve Talents Program of Chongqing (to HY).

## Conflict of Interest

The authors declare that the research was conducted in the absence of any commercial or financial relationships that could be construed as a potential conflict of interest.

## Publisher’s Note

All claims expressed in this article are solely those of the authors and do not necessarily represent those of their affiliated organizations, or those of the publisher, the editors and the reviewers. Any product that may be evaluated in this article, or claim that may be made by its manufacturer, is not guaranteed or endorsed by the publisher.
